# Cancer drug resistance in multiple myeloma

**DOI:** 10.20517/cdr.2022.32

**Published:** 2022-03-25

**Authors:** Fatih M. Uckun

**Affiliations:** ^1^Departmens of Oncology and Developmental Therapeutics, Ares Pharmaceuticals, St. Paul, MN 55110, USA.; ^2^Departmens of Translational Oncology, Reven Pharmaceuticals, Westminster, CO 80234, USA.

## INTRODUCTION

Multiple myeloma (MM) is the second most common hematologic malignancy after non-Hodgkin’s lymphoma. Intrinsic and acquired drug resistance of cancer cells to standard drugs is a major obstacle for a more successful survival outcome of MM patients treated on contemporary clinical protocols. The primary purpose of this special issue on “Drug Targets and Resistance Mechanisms in Multiple Myeloma” was to collect new and transformative information regarding new insights about the mechanisms of drug resistance in MM and the role of the tumor microenvironment in treatment failures.

## MAIN TEXT

Overcoming inherent and acquired drug resistance of MM cells, especially in the context of the immunosuppressive tumor microenvironment (TME), remains a major challenge to effective therapy of high-risk or relapsed/refractory (R/R) MM^[1-15]^. Amplified expression of drug transporters P-glycoprotein (P-gp/ABCB1) and multidrug-resistance-associated protein 1 (MRP1/ABCC1) have been implicated in the resistance of MM cells to drugs that are known substrates for these proteins, such as melphalan, dexamethasone, and anthracyclines [Table 1]^[16-18]^. Some studies have indicated that abundant expression levels of the lung resistance protein (LRP), another drug transporter protein, may also confer resistance to chemotherapy drugs and proteasome inhibitors (PIs) in MM^[16-18]^. It remains to be seen if a potent and safe inhibitor of these drug transporters can be identified and clinically leveraged to overcome drug resistance in MM. Several humoral and cellular components of the TME facilitate the immune evasion and subsequent expansion of drug-resistant MM clones, such as myeloid-derived suppressor cells (MDSCs), regulatory T-cells, and MM-derived cytokines including TGF-β, and IL-6, and IL-10^[4]^. MYC and hepatocyte growth factor/c-MET signaling networks have also been identified as potential contributors to cancer drug resistance in MM^[15]^. Patients with triple-class-refractory MM whose cells exhibit triple-class resistance to PIs, immunomodulatory drugs (IMiDs), and monoclonal antibodies (MoAb) have an OS of < 6 months emphasizing the urgency of this unmet medical need^[19-20]^.

**Table 1:** Possible Mechanisms of Cancer Drug Resistance in Multiple Myeloma

**Therapeutics**	**Resistance Mechanism**
IMiD	Mutations of proteins in CRBN-IK axis associated with deficient expression
PI	Impaired binding to PSMB5 due to mutations; MIF overexpression; LRP abundance; epigenetic reprogramming; DNA hypermethylation in an active intronic CRBN enhancer
Chemo	Amplified expression of P-gp/ABCB1 and MRP1/ABCC1
MoAb	Low expression level of target antigen; upregulation of the complement inhibitors CD55 and CD59
DEX	GR mutations and deficiency; amplified expression of P-gp/ABCB1 and MRP1/ABCC1

DEX: Dexamethasone; IMiD: immuno-modulatory drug; PI: proteasome inhibitor; Chemo: chemotherapy drugs; MIF: migration inhibitory factor; LRP: lung resistance protein; P-gp: P-glycoprotein; MRP1: multidrug-resistance-associated protein 1.

IMiDs such as thalidomide, lenalidomide, and pomalidomide trigger cereblon (CRBN)-mediated ubiquitination and degradation of important regulatory proteins that contribute to the expansion of drug-resistant clones, including the transcription factor Ikaros^[18]^. They also augment the proliferation and effector function of cytotoxic T-cells (CTLs) and natural killer (NK) cells while inhibiting immunosuppressive T-regs. Unfortunately, deficient expression of the target CRBN, as reported for MM cells with certain mutations of the *cereblon *gene or DNA hypermethylation in an active intronic CRBN enhancer, occurs in almost one-third of the patients with R/R MM and causes resistance to the IMiDs [Table 1]^[21,22]^. CRBN-independent resistance to IMiDs has also been reported^[1,4]^. Ongoing research will explore if the clinical benefit of IMiDs could be augmented by using them in combination with CRBN E3-ligase modulators (e.g., iberdomide) to accelerate the degradation of Ikaros transcription factor proteins IKZF1/IKZF3^[1,4]^.

In contemporary induction protocols for MM, IMiDs are often combined with PIs such as bortezomib and Carfilzomib^[5,20]^. PIs trigger apoptotic death of MM cells by contributing to an exaggerated unfolded protein response (UPR) pathway and ER stress^[1,4]^. They further impair the survival-promoting interactions between MM cells and stromal elements in the bone marrow microenvironment, and they promote the immunogenic death of damaged MM cells^[1,4]^. Impaired binding of PIs to the target proteasome β5 subunit (PSMB5) has been associated with PI resistance and can occur due to mutations of the encoding gene that cause conformational alterations at the binding region [Table 1]^[23,24]^. MicroRNAs (miRNAs) play important roles in mRNA silencing and regulation of gene expression in MM cells^[25]^. CD47 antigen is overexpressed in MM, likely due to miR155 down-regulation, and its abundance was associated with a poor prognosis^[26-28]^. Recent studies have implicated CD47 in PI resistance of MM cells^[28]^. In view of the promising early clinical experience in patients with myeloid malignancies, evaluation of the anti-CD47 MoAb Magrolimab in R/R MM patients would also seem warranted^[4]^. Notably, CD47-targeting with synthetic micro-RNA miR-155 overcame bortezomib resistance and induced phagocytosis as well as apoptosis of MM cells by causing loss of CD47^[28]^. Likewise, another micro-RNA, miR-218, is decreased in MM and synthetic miR-218 may help overcome PI resistance^[29]^. In the future, nanoformulations of synthetic miR155 and miR-218 could be used for the resensitization of resistant MM cells to PI. Another emerging new strategy to overcome PI resistance involves the targeting of the macrophage migration inhibitory factor, which has been shown to render MM cells resistant to PI-induced apoptosis^[30]^. Clinical biomarker studies have demonstrated that the high-level expression of this target is associated with poor prognosis and survival in MM^[30]^.

BCL-2 is a predominant anti-apoptotic protein in B-lineage lymphoid malignancies, including MM. Venetoclax is a BCL-2 homology 3 (BH3)-mimetic that disrupts the association of the proapoptotic BH3-only proteins such as BIM and BID with BCL-2^[31]^. BCL-2 inhibition by Venetoclax could theoretically damage chemotherapy-resistant MM cells by inhibiting the amino acid metabolism and reducing oxidative phosphorylation like its effects on leukemic cell populations^[1,32,33]^. Venetoclax exhibited meaningful single-agent activity in R/R MM patients, especially those with a t(11;14) translocation^[34]^. A combination of Venetoclax plus Bortezomib and dexamethasone was more effective than placebo plus bortezomib and dexamethasone in patients with R/R MM, albeit with higher toxicity due to infections^[35]^. Besides BCL-2, MCL-1 is also an important survival-promoting anti-apoptotic protein for MM cells, and inhibitors of MCL-1 such as AMG-176 and MIK665 have been developed as potential anti-MM drugs that could be combined with other apoptosis-promoting anti-MM drug candidates including Venetoclax^[36]^.

A recent prospective clinical study employed longitudinal single-cell RNA-sequencing (scRNA-seq) to study the mechanism and dynamics of drug resistance in MM^[37]^. An enzyme of the UPR pathway, peptidylprolyl isomerase A, was identified as a new molecular target demonstrating the clinical potential of this new strategy in identifying clinically relevant new therapeutic targets for overcoming cancer drug resistance in MM^[37]^. Another important strategy for further identification of new molecular targets contributing to cancer drug resistance in MM is deep measurable residual disease (MRD) profiling, which is based on the characterization of MRD clones using flow cytometry in combination with whole-exome sequencing^[38]^.

Precision medicines as well as biotherapeutic agents, including therapeutic monoclonal antibodies such as the anti-CD38 MoAb Daratumumab and isatuximab, and the anti-signaling lymphocyte activation marker F7, antibody elotuzumab, antibody-drug conjugates, and bispecific antibodies (BiAb) have been developed to damage drug-resistant MM clones as well as alter the immunosuppressive bone marrow microenvironment with some very promising clinical data regarding their clinical impact potential^[1,4,39]^. T-cell redirecting BiAb and bispecific T-cell engagers (BiTES) targeting CD38, the orphan G protein-coupled receptor GPRC5D, and the B-cell maturation antigen (BCMA)/CD269 on MM cells and CD3 antigen on T-cells facilitate the CTL-mediated destruction of drug-resistant MM cells in cytolytic synapses^[4,40]^. They showed promising single-agent activity in early clinical trials of R/R MM patients, and risk mitigation strategies have been identified for their potentially serious side effects such as cytokine release syndrome and neurotoxicity^[4,40]^. BCMA-targeting cellular immunotherapy platforms using chimeric antigen receptor (CAR)-T cells or NK cells have also been developed with documented objective clinical responses in single-agent trials^[1,4,6-9,40]^.

Another area of active clinical research to improve the outcome of cancer drug-resistant MM is related to efforts for overcoming the immunosuppressive TME^[4,40]^. BiAb and anti-CD38 MoAb are capable of dual targeting of both MM cells and immunosuppressive elements of the TME such as the MDSC, and are being explored as potential therapeutic platforms^[40]^.

Each of the new modalities designed to mitigate or overcome cancer drug resistance in MM has faced inherent and acquired resistance mechanisms^[21]^. For example, soluble BCMA renders MM cells resistant to the cytolytic actions of BCMA-directed BiAb/BiTES and CAR-T cells by serving as a competing target for these biotherapeutic platforms. It remains to be seen if this resistance can be overcome by using a γ-secretase inhibitor to reduce the γ-secretase mediated production of soluble BCMA. It will be important to develop multi-modality combination regimens to minimize the risk of escape by drug-resistant MM clones as well as the emergence of MM clones refractory to the new agents with promising activity^[1,4,13,20,21,35,40-45]^. The timely definition of optimal strategies for overcoming the cancer drug resistance in MM will require randomized adaptive clinical trials with multiple parallel cohorts, each evaluating a promising new treatment strategy.

## DECLARATIONS

### Copyright

© The Author(s) 2022.

## References

[1] Uckun FM, Qazi S, Demirer T, Champlin RE (2019). Contemporary patient-tailored treatment strategies against high risk and relapsed or refractory multiple myeloma. EBioMedicine.

[2] Minnie SA, Hill GR (2020). Immunotherapy of multiple myeloma. J Clin Invest.

[3] Martínez-martín S, Soucek L (2021). MYC inhibitors in multiple myeloma. Cancer Drug Resist.

[4] Uckun FM (2021). Overcoming the Immunosuppressive tumor microenvironment in multiple myeloma. Cancers (Basel).

[5] Voorhees PM, Kaufman JL, Laubach J (2020). Daratumumab, lenalidomide, bortezomib, and dexamethasone for transplant-eligible newly diagnosed multiple myeloma: the GRIFFIN trial. Blood.

[6] Topp MS, Duell J, Zugmaier G (2020). Anti-B-cell maturation antigen BiTE molecule AMG 420 induces responses in multiple myeloma. J Clin Oncol.

[7] Fayon M, Martinez-Cingolani C, Abecassis A (2021). Bi38-3 is a novel CD38/CD3 bispecific T-cell engager with low toxicity for the treatment of multiple myeloma. Haematologica.

[8] Alhallak K, Sun J, Jeske A (2021). Bispecific T cell engagers for the treatment of multiple myeloma: achievements and challenges. Cancers (Basel).

[9] Berdeja JG, Madduri D, Usmani SZ (2021). Ciltacabtagene autoleucel, a B-cell maturation antigen-directed chimeric antigen receptor T-cell therapy in patients with relapsed or refractory multiple myeloma (CARTITUDE-1): a phase 1b/2 open-label study. Lancet.

[10] Usmani SZ, Garfall AL, van de Donk NWCJ (2021). Teclistamab, a B-cell maturation antigen × CD3 bispecific antibody, in patients with relapsed or refractory multiple myeloma (MajesTEC-1): a multicentre, open-label, single-arm, phase 1 study. Lancet.

[11] Berdeja JG, Krishnan AY, Oriol A (2021). Updated results of a phase 1, first-in-human study of talquetamab, a G protein-coupled receptor family C group 5 member D (GPRC5D) × CD3 bispecific antibody, in relapsed/refractory multiple myeloma (MM). J Clin Oncol.

[12] Gozzetti A, Ciofini S, Sicuranza A (2022). Drug resistance and minimal residual disease in multiple myeloma. Cancer Drug Resist.

[13] Moscvin M, Ho M, Bianchi G (2021). Overcoming drug resistance by targeting protein homeostasis in multiple myeloma. Cancer Drug Resist.

[14] Black H, Glavey S (2021). Gene expression profiling as a prognostic tool in multiple myeloma. Cancer Drug Resist.

[15] Giannoni P, de Totero D (2021). The HGF/c-MET axis as a potential target to overcome survival signals and improve therapeutic efficacy in multiple myeloma. Cancer Drug Resist.

[16] Pinto V, Bergantim R, Caires HR, Seca H, Guimarães JE, Vasconcelos MH (2020). Multiple myeloma: available therapies and causes of drug resistance. Cancers (Basel).

[17] Mynott RL, Wallington-Beddoe CT (2021). Drug and solute transporters in mediating resistance to novel therapeutics in multiple myeloma. ACS Pharmacol Transl Sci.

[18] Mynott RL, Wallington-Beddoe CT (2021). Inhibition of P-glycoprotein does not increase the efficacy of proteasome inhibitors in multiple myeloma cells. ACS Pharmacol Transl Sci.

[19] Gandhi UH, Cornell RF, Lakshman A (2019). Outcomes of patients with multiple myeloma refractory to CD38-targeted monoclonal antibody therapy. Leukemia.

[20] Moreau P, Kumar SK, San Miguel J (2021). Treatment of relapsed and refractory multiple myeloma: recommendations from the International Myeloma Working Group. Lancet Oncol.

[21] Davis LN, Sherbenou DW (2021). Emerging therapeutic strategies to overcome drug resistance in multiple myeloma. Cancers (Basel).

[22] Haertle L, Barrio S, Munawar U (2021). Cereblon enhancer methylation and IMiD resistance in multiple myeloma. Blood.

[23] Wallington-Beddoe CT, Sobieraj-Teague M, Kuss BJ, Pitson SM (2018). Resistance to proteasome inhibitors and other targeted therapies in myeloma. Br J Haematol.

[24] Barrio S, Stühmer T, Da-Viá M (2019). Spectrum and functional validation of PSMB5 mutations in multiple myeloma. Leukemia.

[25] Puła A, Robak P, Robak T (2021). MicroRNA in multiple myeloma - a role in pathogenesis and prognostic significance. Curr Med Chem.

[26] Muz B, Kusdono HD, King J (2017). Targeting CD47 As a novel therapeutic strategy in multiple myeloma. Blood.

[27] Kim D, Wang J, Willingham SB, Martin R, Wernig G, Weissman IL (2012). Anti-CD47 antibodies promote phagocytosis and inhibit the growth of human myeloma cells. Leukemia.

[28] Rastgoo N, Wu J, Liu A (2020). Targeting CD47/TNFAIP8 by miR-155 overcomes drug resistance and inhibits tumor growth through induction of phagocytosis and apoptosis in multiple myeloma. Haematologica.

[29] Chen H, Cao W, Chen J (2021). miR-218 contributes to drug resistance in multiple myeloma via targeting LRRC28. J Cell Biochem.

[30] Wang Q, Zhao D, Xian M (2020). MIF as a biomarker and therapeutic target for overcoming resistance to proteasome inhibitors in human myeloma. Blood.

[31] Bajpai R, Matulis SM, Wei C (2016). Targeting glutamine metabolism in multiple myeloma enhances BIM binding to BCL-2 eliciting synthetic lethality to venetoclax. Oncogene.

[32] Lagadinou ED, Sach A, Callahan K (2013). BCL-2 inhibition targets oxidative phosphorylation and selectively eradicates quiescent human leukemia stem cells. Cell Stem Cell.

[33] Jones CL, Stevens BM, D’Alessandro A (2018). Inhibition of amino acid metabolism selectively targets human leukemia stem cells. Cancer Cell.

[34] Kumar S, Kaufman JL, Gasparetto C (2017). Efficacy of venetoclax as targeted therapy for relapsed/refractory t(11;14) multiple myeloma. Blood.

[35] Kumar SK, Harrison SJ, Cavo M (2020). Venetoclax or placebo in combination with bortezomib and dexamethasone in patients with relapsed or refractory multiple myeloma (BELLINI): a randomised, double-blind, multicentre, phase 3 trial. Lancet Oncol.

[36] Bolomsky A, Miettinen JJ, Malyutina A (2021). Heterogeneous modulation of Bcl-2 family members and drug efflux mediate MCL-1 inhibitor resistance in multiple myeloma. Blood Adv.

[37] Cohen YC, Zada M, Wang SY (2021). Identification of resistance pathways and therapeutic targets in relapsed multiple myeloma patients through single-cell sequencing. Nat Med.

[38] Goicoechea I, Puig N, Cedena MT (2021). Deep MRD profiling defines outcome and unveils different modes of treatment resistance in standard- and high-risk myeloma. Blood.

[39] Sherbenou DW, Su Y, Behrens CR (2020). Potent activity of an anti-ICAM1 antibody-drug conjugate against multiple myeloma. Clin Cancer Res.

[40] Uckun FM (2021). Dual targeting of multiple myeloma stem cells and myeloid-derived suppressor cells for treatment of chemotherapy-resistant multiple myeloma. Front Oncol.

[41] Fontana F, Scott MJ, Allen JS (2021). VLA4-targeted nanoparticles hijack cell adhesion-mediated drug resistance to target refractory myeloma cells and prolong survival. Clin Cancer Res.

[42] Byrgazov K, Kraus M, Besse A (2021). Up-regulation of multidrug resistance protein MDR1/ABCB1 in carfilzomib-resistant multiple myeloma differentially affects efficacy of anti-myeloma drugs. Leuk Res.

[43] Chari A, Vogl DT, Gavriatopoulou M (2019). Oral Selinexor-dexamethasone for triple-class refractory multiple myeloma. N Engl J Med.

[44] Innao V, Rizzo V, Allegra AG, Musolino C, Allegra A (2021). Promising anti-mitochondrial agents for overcoming acquired drug resistance in multiple myeloma. Cells.

[45] Federico C, Alhallak K, Sun J (2020). Tumor microenvironment-targeted nanoparticles loaded with bortezomib and ROCK inhibitor improve efficacy in multiple myeloma. Nat Commun.

